# The Coronaviruses of Animals and Birds: Their Zoonosis, Vaccines, and Models for SARS-CoV and SARS-CoV2

**DOI:** 10.3389/fvets.2020.582287

**Published:** 2020-09-24

**Authors:** Ahmed M. Alluwaimi, Ibrahim H. Alshubaith, Ahmed M. Al-Ali, Salah Abohelaika

**Affiliations:** ^1^Department of Microbiology and Parasitology, College of Veterinary Medicine, King Faisal University, AlAhsaa, Saudi Arabia; ^2^Department of Environmental Health, AlAhsa Municipality, AlAhsa, Saudi Arabia; ^3^Central Biotechnology Laboratory, Veterinary Teaching Hospital, College of Veterinary Medicine, King Faisal University, AlAhsaa, Saudi Arabia; ^4^Clinical Pharmacology Department, Qatif Central Hospital, Ministry of Health, Qatif, Saudi Arabia

**Keywords:** coronaviruses, SARS-CoV, SARS-CoV-2, BCoV, FCoV, zoonosis, MERS-CoV

## Abstract

The viruses of the family *Coronaviridae* are ubiquitous in nature due to their existence in a wide spectrum of mammals and avian species. The coronaviruses, as RNA viruses, exist as quasispecies because of their high rate of mutations. This review elaborates on the pathogenesis and the developed vaccines of most of the ubiquitous coronavirus' diseases, mainly bovine, dromedary camel, porcine, feline, canine, and avian coronaviruses. The review emphasizes the significant setbacks in the full exploitation of most of the pathogenesis of the coronavirus' diseases, raising the prospect of effective vaccines for these diseases. The therapeutical trials for the treatment of SARS-CoV2 and the setbacks of these trials are also addressed. The review draws attention to the lessons accumulated from the large number of studies of the pathogenesis of animals and birds' coronaviruses and their vaccines, particularly the bovine, feline, and avian coronaviruses. The lessons drawn from the studies will have an immense influence on how the human coronaviruses pathogenesis and vaccine development will proceed. In addition, the extensive efforts to designate suitable animal models to study the lately emerged human coronaviruses are one of the invaluable contributions carried out by veterinarian scientists. Finally, factors and determinants that contribute to the possibility of emerging new coronavirus zoonotic disease are elaborated on and a call goes out to urge transdisciplinary collaboration in the implementation of the “One Health” concept.

## Introduction

Coronaviruses (CoVs) are a family of enveloped single-stranded RNA viruses of medical and veterinary importance that infect mammals and birds, causing respiratory or enteric diseases (1). CoVs are members of the subfamily *Coronavirinae* in the family *Coronaviridae* and *Nidovirales*. The most distinctive feature of *Coronaviridae* is the genomic size, having as they do the largest genomes among all RNA viruses (26.4–31.7 kb in length) with a G+C content varying from 32 to 43% (2). In 1937, Avian Infectious Bronchitis Virus (IBV) was first isolated from an outbreak in chicken flocks. Since then, related CoVs have been discovered with subsequent viral isolations in rodents, domestic animals, and humans (3). The first human CoV was isolated in the 1960s from nasal discharges of patients suffering from the common cold (4). The two human CoVs (HCoVs)—HCoV-OC43 and HCoV-229E—are estimated to be involved in about 30% of common colds. Since then, more knowledge has accumulated through extensive studies of HCoV-229E and HCoV-OC43 (5). It was believed that infection with HCoVs was mild until the outbreak of Severe Acute Respiratory Syndrome-Coronavirus (SARS-CoV). The outbreak of SARS-CoV, in 2003, was one of the most devastating spillovers in current history, infecting over 8000 people and having a crude case fatality of ~10% (6, 7). A decade later, another zoonotic infection emerged in the Middle East region, the Middle East Respiratory Syndrome-Coronavirus (MERS-CoV), which caused a spillover in 2012 that resulted in a persistent epidemic in the Arabian peninsula and sporadic spreading to the rest of the world (8, 9). The MERS-CoV infection of humans causes fatal severe pneumonia (8), with a case mortality of 35% (10). The 2019 novel HCoV (2019-nCoV), which was subsequently renamed SARS-CoV2 (11), is a newly emerged human CoV that has resulted in a global and ongoing pandemic that has claimed more than 710,110 lives and infected more than 18,895,712 people as of August 5, 2020 (12).

It is strongly believed that HCoVs have a zoonotic origin from bats, mice, or domestic animals (13, 14). Indeed, more specifically, ample evidence suggests that the evolutionary origin of all HCoVs lies in bats, which are well-adapted and non-pathogenic but show great genetic diversity. Tracing the zoonotic origins of HCoVs provides a framework to understand the natural history, driving force, and restrictive factors of cross-species transmission (14).

### CoV Taxonomy and Phylogeny

The name of the CoV family comes from the spiky crown (or *corona* in Latin) on its outer surface, visible when viewed through an electron microscope. In the current taxonomy of viruses, the order, family, genus, and species are universally used to organize all diversity of viruses within a hierarchical system (15, 16). To overcome the complexity of similarities found between virus groups, a subfamily rank is added. Viruses are assigned to a particular taxonomic position according to results of the comparative analysis of selected properties, characterizing different aspects of the genome and virion structures and the replication strategy of viruses (17). Accordingly, the classification of CoVs was largely based on cross-reaction to the viral protein, but the classification is currently based on comparative sequence analyses of replicative proteins (17).

The *Coronaviridae* family is made up of the genera Coronavirus and Torovirus (18). Toroviruses were originally proposed to form a new family separate from CoVs (19). However, comparative data analyses led to its recognition as a genus within the *Coronaviridae* (20, 21). CoVs occupy the subfamily of *Coronavirinae* within the family *Coronaviridae*, order *Nidovirales*. Depending on their antigenic and genetic properties, CoVs are classified into three groups (22). The major group of CoVs comprises porcine Transmissible Gastroenteritis Virus (TGEV), Feline Coronavirus (FCoV), Canine Coronavirus (CCoV), HCoV-229E, and Porcine Epidemic Diarrhea Virus (PEDV). The second group consists of Murine Hepatitis Virus (MHV), Bovine Coronavirus (BCoV), HCoV-OC43, Porcine Hemagglutinating Encephalomyelitis Virus (HEV), Rat Coronavirus (RtCoV), and Equine Coronavirus (ECoV). The third group comprises IBV, Turkey Coronavirus (TCoV), and Pheasant Coronavirus (23).

Recently, these three groups have been reclassified into four genera, *Alphacoronavirus, Betacoronavirus, Gammacoronavirus*, and *Deltacoronavirus*. The first two genera include only mammalian CoVs, with human CoVs found in each of these groups, while the other two genera are confined to avian CoVs (24).

### CoVs–Host Interaction

CoVs cause severe diseases in different animals such as dogs, cats, pigs, chicken, cows, camels, and humans, as with the recently emerged spillover caused by COVID-19 (SARS-CoV2), which broke out in China's Wuhan seafood market in December 2019 (25). Epithelial cells are the first line of host defenses against viral infection. CoVs pathogenesis is characterized by diffuse alveolar damage to the lungs, epithelial cell proliferation, and elevation in macrophages. Moreover, CoV infections are associated with multinucleate giant cells, infiltration of macrophage, or epithelial cells known as putative syncytium-like formation (26). Stimulated macrophages in the alveoli release proinflammatory cytokines as an important anti-CoVs strategy (26). In the first 2 weeks of the infection, the virus replicates slowly in macrophages. Ten to 21 days after primary infection, a dramatic increase in the macrophage infiltration and virus replication occurs (27).

Viral entry into the host cells is facilitated by the binding of the spike (S) protein to the cell surface receptors (26, 28). In general, the S protein of CoVs is functionally divided into the S1 subunit (responsible for receptor binding) and the S2 subunit (responsible for cell membrane fusion) (29). Both the N-terminal and the C-terminal domains of the S1 subunit can bind to host receptor (29).

Recently, it was shown that SARS-CoV2 uses angiotensin-converting enzyme-2 (ACE2) as a port of entry, an important finding to understand SARS-CoV2 transmissibility and pathogenicity (30, 31). In addition, recent evidence indicates that the spikes of SARS-CoV2 harbor a predilection site for the polybasic cleavage site, the site of the proteolytic excision, of the furin enzyme (32, 33). The furin cleavage site of the S protein is located in the boundary of the S1 and S2 moieties of the receptor binding site of the S protein (33). Four amino acids (PRRA) were determined as the cleavage targets of the furin: pro681 (p681), Arg682 (R682), Arg683 (R683), and Ala684 (A684). The furin was found to be essential for the cleavage of the human immunodeficiency virus (HIV) glycoprotein (gp) 160 to gp120 and gp41 (34). In contrast, the furin cleavage of the S protein of SARS-CoV2 has no modification on the three-dimensional structure of the S protein (33). The furin activity merely induces rearrangement of the viral binding site, which facilitates the viral attachment to the ACE2 receptor and/or its entry (33). The exact effect of the furin cleavage on the viral surface protein is not fully known; however, it has been speculated that this polybasic cleavage is crucial for the viral transmission and pathogenesis (32). In conclusion, the furin cleavage site between S1 and S2 is considered a vital evolution step in SARS-CoV2 crossing species barriers to become a full human pathogen (32).

Many betacoronaviruses utilize aminopeptidase N (APN) as the entry receptors, but CoVs are also able to utilize other different receptors, such as, for example, MERS-CoV, which binds to dipeptidyl-peptidase 4 (DPP4) to gain entry into human cells (30, 35). DPP4 is predominantly expressed in the upper respiratory tract (36). HCoV-NL63, on the other hand, uses ACE2 as the port of entry, while MHV enters through carcinoembryonic antigen-related cell adhesion molecules (CEACAM) (CD66a) (26). Other groups of betacoronaviruses, such as BCoV, OC43, and Porcine Hemagglutinating Encephalomyelitis Virus (PHEV), bind to 9-O-acetylated sialic acid-containing receptors (26). However, other groups of alphacoronaviruses, such as HCoV-229E, Feline Infectious Peritonitis Virus (FIPV), and TGEV, interact with aminopeptidase N (APN), a zinc-binding protease (26, 37).

In addition to the S protein, some groups of betacoronaviruses have an additional membrane protein, hemagglutinin esterase (HE). The exact function of HE is not fully disclosed, but it has been speculated that HE aids in viral entry and/or pathogenesis *in vivo* (26).

## Animal CoV Diseases

### Feline CoVs

Feline Enteric Coronavirus (FECV) is a virulent biotype of FCoV in domestic cats, and it is the hypervirulent biotype precursor of FIPV that causes fatal diseases in cats. FIPV is classified into two forms, wet and dry sarcoidosis (27). FIPV initially replicates in the epithelial cells of the pharynx or in the intestinal tract (jejunum). The acute phase of infection and virus replication in macrophages are associated with viremia and the rapid spread of the virus in the abdomen and thorax, resulting in lethal inflammation, and is occasionally associated with neurological disorders (26, 27, 38).

### Canine CoVs (CCoVs)

CCoV is genetically related to CoVs of pigs and cats. CCoV is divided into two genotypes, CCoV type-I (CCoV-I) and type-II (CCoV-II) (37). The main predilection site of CCoV infection is the gastrointestinal tract. Canine Enteric Coronavirus (CECoV) infection is associated with high morbidity and low mortality. The main route of the viral transmission is via the oral route (38, 39). TGEV is antigenically related to CCoV (38). The virus infects the epithelial cells of the small intestine, causing gastroenteritis and leading to fatal diarrhea, and occasionally infects the upper respiratory tract (26).

### Porcine CoVs

Porcine Respiratory Virus (PRCoV) is a variant of TGEV that binds to epithelial cells of the lungs, causing antigen aggregation in pneumocytes and alveolar macrophages, which result in interstitial pneumonia (26). On the other hand, PHEV was derived originally from a bat virus and has developed in certain rodents as intermediate hosts (38). PHEV was first circulated among cattles and then it has been developed from bovine CoV (BCoV) to become porcine CoV (BCoV) (38). The newly emerged porcine CoV in Europe, PEDV, causes significant morbidity and mortality of piglets due to enteric infection, possibly resulting in nervous system infection (encephalitis) (26, 40).

### Bovine CoVs (BCoVs)

BCoV infection leads to financial losses in the cattle industry and infection could be extended to camel herds. BCoV infects the respiratory and gastrointestinal tracts, leading to severe diarrhea in calves, with or without respiratory disease. High mortality rates are due to the bloody diarrhea that results from the destruction of the small and large intestinal villi (41), suggesting a lack of long-term mucosal immunity after infection (38, 40). In adult cattle, the infection causes severe or fatal infection when combined with other factors, mainly stress due to shipping or fever pneumonia or coinfections with other secondary respiratory pathogens (40, 41).

### Bird CoVs

Group 3 of CoVs contains viruses that infect a wide range of fowl species (37). IBV is a contagious virus causing high economic losses to the poultry industry (42). It spreads by aerosols because of its replication in the upper respiratory tract and the epithelial surfaces of the alimentary canal, as well as kidneys, gonads, and bursa, causing a dramatic drop in egg production (26, 37).

### Dromedary Camel CoVs

MERS-CoV causes mild symptoms in dromedary camels in natural or experimental infection (36). Experimental infection of camels with MERS-CoV reveal restricted replication to the upper respiratory tract, particularly in the epithelium of the nasal turbinate (43). MERS-CoV mainly causes fever, nasal and lachrymal discharge, coughing, sneezing, and loss of appetite (36). Camel calves have a tendency to shed a higher level of MERS-CoV than adults (44).

### Murine Hepatitis Virus (MHV)

MHV causes respiratory, enteric, hepatic, and neurologic infections in mice (26).

## Human CoVs

Prior to the emergence of SARS-CoV, the two prototypes of human CoVs—OC43 and 229E CoV—were primarily associated with the common cold (26). The human CoV variants, NL63, which was isolated in late 2004, and HKU1, which was isolated in January 2005 (31), were found to be associated with mild respiratory or enteric diseases in humans (30). The most infectious human CoVs that cause severe infection are SARS-CoV and MERS-CoV (31, 41). Phylogenic studies have speculated that SARS-CoV is of a bat origin that was then developed in civet cats as an intermediate host (35). SARS-CoV uses ACE2 as a binding receptor to infect the lung epithelial cells. SARS-CoV causes systemic disease with extrapulmonary dissemination accompanied with viral shedding in different body secretions (26). The viral shedding elevates after 6 days until it reaches a peak at 12 to 14 days after the onset of the disease (6).

MERS-CoV was recognized as a newly emerged zoonotic disease developed in dromedary camels as an intermediate host (43). A survey of camel herds indicated that 90% of dromedary camels were seroconverted to MERS-CoV (44). Similar to SARS-CoV, MERS-CoV phylogenic analysis indicates that the virus is an ancestor of bat CoV (30).

In humans, MERS-CoV infects both the upper and lower respiratory tract, with a higher concentration of the MERS-CoV RNA in the lower respiratory tract (36). Lymphocytopenia, as a result of apoptosis, was reported frequently in patients, particularly the phenotypes CD3^+^, CD4^+^, and CD8^+^ T-lymphocytes (45).

The other newly emerged highly pathogenic human zoonotic disease, SARS-CoV2, is characterized by a high frequency of asymptomatic infection of the respiratory tract and to some extent shows enteric symptoms. The major symptoms of the SARS-CoV2 infection are limited viremia, lymphocytopenia, pneumonia, and lymphocyte infiltration (25, 38). Complications in cases suffering from severe symptoms are attributed to the immunopathological responses, particularly copious cytokine production (known as cytokines storm syndrome) (46).

The bat Rhinolophus (horseshoe bat) is speculated as the harbor of the ancestor of SARS-CoV2. RNA sequence analysis advocates these speculations due to the close homology between the SARS-CoV2 sequence and that isolated from the horseshoe bat (31). Evidence indicates the homology of RNA sequence of SARS-CoV2 to that of the intermediate host Pangolin CoV (47).

## Immune Responses to CoV Infections and Viral Countermeasures

### Innate Immune Responses

Innate immunity is crucial for hindering the CoVs replication and is an essential step in the development of the adaptive immune responses. The responses are initiated by the capability of the pattern recognition receptors (PRRs), molecules on the cell surface and in the cytoplasm to sense certain sequences of the viral genome and particular proteins, known as pathogen-associated molecular patterns (PAMPs) (48, 49). The well-documented CoVs PAMPs are intermediate double-stranded RNA and the 5′-triphosphate-bearing RNA, which trigger the intracellular PRRs, retinoic acid-inducible-I receptor (RIG-I) and melanoma differentiation-associated protein 5 (MAD-5) (48, 49). The toll-like receptors (TLRs) TLR-3, TLR-7, and TLR-8 are also triggered by the CoV nucleic acids. The viral M and N proteins are the important PAMPs that are sensed by the TLR-2 and TLR-4. Activation of the PRRs initiate the expression of interferon-regulatory factor 3 and 7 (IFN-3 and IFN-7) and NFκB pathway, which prime a set of macromolecular pathways leading to the expression of antiviral interferon-stimulated genes (ISGs) (48, 49). Consequent to the activation of the ISGs, we get the copious production of type-I IFNs and other proinflammatory cytokines such as interleukin (IL)-1, IL-6, IL-8, and tumor necrosis factor-β (TNF-β). The innate responses due to the activation of ISGs result in drastic interference with most of the viral replication machineries. Despite the importance of proinflammatory cytokines in creating unfavorable condition for viral replication, the secreted cytokines are accompanied with lethal pathological consequences, known as “cytokine storms” (46, 48, 49). Nonetheless, CoVs have evolved an evasive mechanism that could overcome the innate responses, particularly interference with ISGs activation. The CoVs nonstructural proteins (NSP), like NSP1 of SARS-CoV, act as a major antagonist to IFN signaling by hindering the pathways initiated by type-I IFN (49).

### Adaptive Immune Responses

The specific cellular immune responses to CoVs are associated with vast activation of CD8^+^, T-cytotoxic lymphocyte and CD4^+^, T-helper lymphocyte (48, 50). Dendritic cells (DCs) are important antigen-processing cells that trap, process, and present viral antigens to T-lymphocytes. The DCs resident in the respiratory tract acquire the CoVs antigen(s) and migrate to the regional lymph nodes (mediastinal and cervical) to prime T-lymphocytes (50). CD8^+^ cytotoxic T-lymphocyte represents 80% of the major effector cells that are recruited to the pulmonary interstitium to clear the viral persistence by cytotoxic mechanism (50).

CD4^+^ T-lymphocytes, however, in addition to B-lymphocyte activation for antibody production, mediate pro-inflammatory responses by production of IL-17, which recruit monocytes and neutrophils as well as a wide range of other inflammatory cytokines and chemokines, like IL-1, IL-6, IL-8, IL-21, TNF-β, and CCL2 (48). Antibody production is essential for interference with viral attachment in addition to the control of viral persistence. S complementary system could be activated; however, the inflammatory responses aggravated by the activation of C5a and C3a subcomponents inflict lethal consequences rather than protection from the infection (48).

The activated T-lymphocytes generate long-lasting memory cells. CD8^+^ and CD4^+^ memory cells have been detected in patients with acute SARS-CoV infection 4–6 years post recovery. The memory CD8^+^ cells are an active source of IFN-γ and TNF-α with a release of the cytotoxic mediators, perforin, and granzyme B (50). The CD4^+^ memory cells specific for the surface proteins S, M, and N have also been documented. Both CD8^+^ and CD4^+^ memory cells play a crucial role in protecting against reinfection (50). Interestingly, adaptive transfer of CD8^+^ memory cells protect young chicks from IBV infection, but not memory CD4 cells. The existence of B memory cells requires further investigation (50).

CoVs manage various maneuvers to escape the cellular and humoral responses (50). For instance, MERS-CoV infection induces a high level of apoptosis, which leads to severe lymphopenia. On the other hand, SARS-CoV indirectly interferes with T cells activation by abolishing the maturation of DCs, which are vital for the antigen processing and presentation (48, 50).

## The Vaccines of Animal CoVs and the Vaccination Challenges

Overwhelming efforts have been invested in reaching effective vaccines that resolve the global dilemma of the CoV pandemic. Nevertheless, attempts to license effective CoV vaccines against respiratory infections in humans have not yet been successful (51). However, CoV infection in different domestic animal species, such as cats, dogs, pigs, cattle, and poultry, is managed routinely by vaccination. For instance, the IBV vaccines were the first licensed vaccines to prevent upper respiratory CoV infection in chickens (52, 53). The compiled data generated during the experiments with animals CoV vaccination programs are an invaluable source of designing an effective vaccine to SARS-CoV2 (54). Hence, CoV vaccines of veterinary applications highlight numerous successes, potentially allowing the hurdles that are faced in the development of a SARS-CoV2 vaccine to be negotiated (55, 56). Considering the long-term experience gained with animal CoVs, veterinary medicine could participate with major contributions to decipher the origin of SARS-CoV2 and to drive future research in human medicine toward the development of immunogenic and safe vaccines and effective antiviral drugs. The successes and failures encountered with prophylaxis and treatment of animal CoVs, such as FIP, might be useful to address issues related to SARS-CoV2 in the One Health approach.

To combat CoV infections in livestock and poultry, most of the currently available CoV vaccines are inactive or attenuated live vaccines utilizing the advantage of different vaccine technologies such as conventional inactivated, virus vectored, DNA delivery, and mRNA delivery. The majority of vaccines licensed for veterinary applications have been developed for CoVs such as CECoV infection in dogs, PEDV and TGEV infection in pigs, and BCoV in cattle to prevent shipping fever in young calves (57, 58). All of these vaccines have been highly variable in their potency and efficacy. The weakness or failure in vaccination is primarily related to the site of infection and systems affected, which in turn dictate the nature of a vaccine and route of administration (40). For instance, IBV vaccines have low protection, but they ameliorate the severity of the respiratory symptoms and prevent kidney and reproductive tract side effects (40). Parenteral routes in animals do not trigger strong local immunity to induce mucosal immunoglobulin-A (IgA) against enteric or respiratory disease. Mucosal immunity, even if it does not prevent the infection, is effective enough to reduce viral shedding and the severity of the respiratory disease. Furthermore, many CoV local T cell-mediated responses are required for effective protection (59).

Live attenuated vaccines tend to generate better protection than inactivated vaccines. Despite this fact, live vaccines could revert to a virulent strain (58) and have low efficiency in preventing virus shedding. Inactivated vaccines induce partial mucosal immunity; however, they are a good booster of immunity in sows prior to farrowing (54). Other issues that have been taken into consideration while vaccines development are the short duration of protective immunity and poor inactivated vaccine potency. On the other hand, these types of vaccines are low cost, especially in the veterinary field, where mass vaccination procedure is practiced (54). The development of recombinant vaccines by using reverse genetics as an attenuation approach has been advocated (54). In addition, recombinant subunit vaccines are also seen as important to produce proteins expressed genetically in modified *Escherichia coli* based on recombinant DNA technology (60).

### CCoV Vaccines

Two types of CCoV vaccines have been developed: the inactivated and the live attenuated vaccines. Dog vaccination with the inactivated CCoV vaccine reduced the level of viral shedding in feces and was effective against experimental challenge (61). Fulker et al. (1995) have stated that a small number of vaccinated dogs (15%) show very mild diarrhea while 80% of the non-vaccinated dogs show severe watery or bloody diarrhea experience with an average of about 10.8 days compared to 1.4 days for vaccinated animals (62). However, the mild status of the disease discouraged the wide application of the vaccine (54). Despite the availability of both inactivated and modified live vaccines against the group 1 virus, their use is not recommended because the infection is mostly mild and self-limiting, with inapparent gastroenteritis, anorexia, fever, and diarrhea. It is believed that protection against CCoV is mainly dependent on the production of IgA, and therefore parenteral vaccination was not favored because it does not produce prime mucosal IgA in the intestine (54).

### FCoV Vaccines

FCoV infection may be of a mild nature or may result in a lethal immune-mediated disease—feline infectious peritonitis (FIP). Feline vaccination encountered the dilemma of generating protective immunity without causing immune-mediated disease. However, cats with preexisting high levels of antibodies against FCoV develop effusive FIP rapidly on challenge. On the other hand, administering antiserum to FCoV before challenge may also enhance the risk of peritonitis. FIP is highly prevalent in young cats between 6 months and 3 years of age (63). In the event of FCoV infection, FIP vaccination with S protein leads to exacerbation of the disease. Stimulation of local IgA was seen as more relevant than IgG stimulation; therefore, administering temperature-sensitive vaccine by the intranasal route is more effective in interfering with the viral invasion (54).

### BCoV Vaccines

Vaccines against BCoV protects against enteric and respiratory disease in young calves. Inactivated vaccines are mainly applied to initiate maternal immunity in pregnant cows (54, 57). BCoV vaccines should stimulate protective mucosal immunity to protect the epithelial lining cells of respiratory system and/or the intestinal tract as the main target of the viral infection. Because of the nature of the disease, which infects calves at an early age, the maternal immunity induced by vaccinating pregnant cows in the third trimester is considered an important control measure to protect calves (52). Another way of protecting calves is to administer the attenuated live vaccine intranasally at day 1, or slightly later, to induce innate responses. Protection against BCoV is attributed mainly to the high levels of serum neutralizing and hemagglutinating antibodies (54).

### IBV Vaccines

High losses in poultry production inflicted by IBV infection is mainly due to the combination of high morbidity and loss of growth performance, accompanied by secondary bacterial infections (38, 53). IBV inactivated and live attenuated vaccines were widely produced. The inactivated vaccines are mainly used as a booster in older, egg-laying chickens. Despite the wide application of the IBV inactivated and live attenuated vaccines, the disease continues to be a major problem for the poultry industry due to the existence of many serotypes. The variations in the surface spike protein denote wide diversity, which leads to poor cross-protection and loss of immunogenicity. Live attenuated IBV vaccines are produced by repeated passage in embryonated eggs, resulting in spontaneous mutations. As a consequence, attenuated viruses have a small number of mutations, which could be associated with the loss of the virulence and/or immunogenicity, with a major risk of reversion to virulence (64). Wide use of the vaccines contributed tremendously to the high variability of IBV through recombination between vaccine strains and the field viruses, as well as selection pressure due to extensive use of the vaccines, which induces partial immunity in the vaccinated birds (38). Hence, the continuous generation of new IBV variants due to the mutation and recombination leads to great difficulty in controlling IBV outbreaks (54).

### Porcine CoVs Vaccines

In swine CoVs, sow vaccination in the gestational stage is seen as essential for producing maternal immunity. Most of the current commercial TGEV vaccines are live attenuated to amplify maternal immunity (65, 66). Most of these vaccines are bi- or trivalent combined with rotavirus, PEDV, and/or *E. coli*. Experimental vaccines include novel DNA vaccines, vectored vaccines, and recombinant vaccines. For example, the porcine adenovirus was used to deliver the TGEV spike protein (67). DNA vaccines were constructed for both PEDV and TGEV (68). Studies have indicated protective immunity in primate rhesus macaques challenged with inactivated whole virus vaccine from China and adenovirus vectored ChAdOx1 nCoV (69). Unfortunately, modified live TGEV vaccines have failed to induce a strong secretory IgA response. The parenteral route also indicated weak IgA response to the killed vaccines (57). The TGEV purified spike proteins were seen as effective antigens in stimulating the mucosal IgA. Both inactivated and live attenuated TGEV and PEDV vaccines were manufactured and have been extensively used in Asia. Although live vaccines could stimulate long-lasting immunity, they remain incapable of preventing viral shedding. Similar to IBV vaccines, the live vaccine strains could become virulent by recombination with circulating strains (58). Subunit or killed vaccines, however, could partially stimulate mucosal immunity, particularly in boosting immunity prior to farrowing (58). Reverse genetics-based modified live attenuated vaccines (MLVs) have been approached in developing the vaccine to the porcine respiratory and reproductive system virus (PRRSV). However, the extensive heterogenicity of the PRRSV strains has influenced the development of effective subunit vaccines (59).

## Animal models for SARS-CoV and SARS-CoV2

Substantial trials have been conducted in different domestic and laboratory animals and birds to assimilate the clinical manifestation of SARS-CoV and SARS-CoV2. However, majority of animal models expressed vast variations in their clinical manifestations. It is essential to envisage the major criteria that denote the animal models as suitable candidates for a given human disease before reviewing the current available data about the experimental trials.

Animals become an appropriate model if they can represent certain aspects of human disease complexity. The animal model candidate does not necessarily mimic the entire human disease complexities rather than specific facets of the disease. There are five criteria for selecting an animal model (70).

(1) Species: the species that are capable of depicting the pathophysiology of the disease close to that reflected in humans are more suitable candidates.
(2) Complexity: it is highly important for the model to reveal the complexity of disease in the utmost detail.
(3) Disease simulation: the simulation of the disease could be subjected to several pathways and principles. Hence, the model should exploit the complexity of the disease through collective pathways and consequences.
(4) Predictivity: a criterion that is mainly applied for assessing the effect of the drug on the final outcome of the disease.
(5) Face validity: this criterion defines the extent of the model in reflecting a symptom or set of symptoms.

Animal models are classified in groups according to their biological, genetic, and pathophysiological requirements (71). They are as follows:

(1) Induced (experimental) models: certain impairments experimentally induced to allow their study.
(2) Spontaneous (genetic, mutant) models: naturally existing genetic variants that allow the study of the side effects of mutation.
(3) Genetically modified models: modification induced by genetic engineering and embryo manipulation.
(4) Negative models: species or breeds that are naturally resistant to certain infectious diseases.
(5) Orphan models: nonhuman species that suffer from certain natural functional disorder(s).

The susceptibility of several domestic and laboratory animals was tested for the expression of the pathoimmunogenicity of SARS-CoV and SARS-CoV2. The following is a list of the most commonly tested animals.

### Cats

The susceptibility of cats to SARS-CoV2 has been the subject of extensive trials for the study of the infection (72). The SARS-CoV2 RNA was detected in the mucosal turbinate of cats inoculated intranasally. The RNA was also detected in soft palates, tonsils, trachea, and small intestine of euthanized cats but was missing in the lung of the infected cats. The uninfected cats kept in cages close to the inoculated cats were permissive to the air drops of the infected cats (72). ELISA test has indicated the seroconversion of the inoculated cats. The histological examination of the euthanized infected cats denoted massive lesion in the nasal and tracheal epithelium and lungs. The outcomes of a study of cat susceptibility to SARS-CoV2 replication in the respiratory system indicated certain reliability; however, young cats appeared to be more susceptible. Uninfected cats, on the other hand, were shown to be susceptible to the infection if they were in close contact with infected cats (72).

Cat susceptibility to SARS-CoV has also been addressed (73). RT-PCR detected RNA in the samples of the pharyngeal swab of SARS-CoV infected cats on day 8 post-infections (Pi). Histologically, the pulmonary lesions were found to be mild.

### Ferrets

The viral RNA was detected in the nasal washes and rectal swabs of intranasally inoculated ferrets. The histological lesions of the low respiratory tract revealed an increase in type II pneumocytes, macrophages, and neutrophils (72).

SARS-CoV2 expressed efficient replication in the ferret digestive tract. The overall clinical signs, viral replication, and the pathological manifestation in ferrets' lower respiratory tract clearly indicated a high susceptibility to SARS-CoV2 (72). Ferret susceptibility to SARS-CoV was similar in the scale of the permissiveness as SARS-CoV2 (73).

Although the viral replication in the respiratory tissue and the manifestation of certain clinical signs indicated that cats are permissive to SARS-CoV infection, the picture of the disease in cats was milder than in ferrets (73). It was speculated that the difference in the susceptibility of ferrets from cats could be attributable to the binding capability of SARS-CoV2 to the receptor of respiratory cells (72). SARS-CoV2 attachment to the epithelial cells of tracheobronchial tissues is facilitated by the attachment to ACE2 receptors. The variation in the susceptibility could be attributed to the difference in a single amino acid of ACE2 of ferrets from that of cats (72). In contrast, six amino acids of the binding site of the S protein of SARS-CoV2 enable the SARS-CoV2 receptor binding site to bind the ACE2 of humans, ferrets, cats, and other species with high affinity (32). The ACE2 amino acids that interact with the viral combining site are Y442, L472, N479, D480, T487, and Y491, whereas the six designated SARS-CoV2 amino acids are L455, F486, Q493, S494, N501, and Y505. On the other hand, SARS-CoV binding site differs from SARS-CoV2 in five of those amino acids (32).

A recent study, however, has attributed the variations in the level of the permissiveness to the SARS-CoV2 infection in humans to the affinity of the intermolecular interaction of ACE2 with the viral binding site of S protein (74). Using homology modeling, it was shown that certain alleles of the ACE2 have significantly low interaction levels with the viral spike protein, which in turn could influence the susceptibility to the SARS-CoV2 infection (74).

### Dogs

The susceptibility of dogs to SARS-CoV2 has also been addressed (72). Although the viral RNA was detected in the dog rectal swab, the viral RNA in organs of euthanized dogs was absent. The seroconversion was only positive in some of the infected dogs (72). The experimental trial on dogs clearly indicated that dogs are of low susceptibility to SARS-CoV2 infection. A follow-up on the naturally infected case of two dogs that contracted the infection from their owner confirmed further low dog susceptibility to SARS-CoV2 infection (75). Despite their seroconversion and detection of SARS-CoV2 RNA in the nasal, oral, and rectal swabs, the clinical signs were very mild and had disappeared by day 14 (75).

### Cynomolgus Macaques and Rhesus Macaques

Cynomolgus and rhesus macaques were inoculated with SARS-CoV to see if they were susceptible to its infection (76). RNA was detected in nasal swabs and in the lung of euthanized macaques. The clinical signs of the inoculated macaques and the pathological lesions in the lungs were very mild (76). Nevertheless, the severity of the lesion in cynomolgus macaques was greater than that in rhesus macaques. Similar findings have been supported by other studies (77).

The susceptibility of cynomolgus macaques to SARS-CoV2 has been evaluated (78). Two groups of cynomolgus macaques—young and old aged—were inoculated intrathecally with field isolates. The overall clinical signs showed a very mild difference in the older-aged group. All the animals were seroconverted by day 14 Pi. SARS-CoV2 RNA was detected in nasal and rectal swabs by day 2 Pi. The autopsy of the experimentally infected macaques clearly indicated the restriction of the infection to the upper and lower respiratory tract. Lesions were mainly the foci of pulmonary consolidation. The viral RNA was also restricted to the respiratory tract whereas no viral RNA was detected in the central nervous system or in the lymphoid tissues.

The overall assessment of experimental infection of cynomolgus macaques with SARS-CoV2 clearly reveals that this nonhuman primate is susceptible to SARS-CoV2 infection (78). The pathological lesions inflicted in the upper and lower respiratory tissues, as well as the viral antigen expression in the pneumocytes type I and II, advocate the possible use of the primate as an animal model for certain aspects of SARS-CoV2 pathological mechanism (78). The results of the experimental infection of rhesus macaques were similar to those recorded in cynomolgus macaques (79).

### Mice Models

Mice are of low susceptibility to SARS-CoV and SARS-CoV2 infection due to the low binding properties to its ACE2 receptor in the respiratory system (80). However, mice that were genetically transduced by human ACE2 gene became susceptible to SARS-CoV infection (81). The transgenic mice model expressing human ACE2 showed high susceptibility and became highly permissive to SARS-CoV infection (81) and SARS-CoV2 (82).

### Other Models

#### Syrian Hamsters

SARS-CoV replication in the respiratory tract of Syrian hamsters led to pulmonary lesions. The hamsters indicated moderate support for viral replication in the respiratory tract, albeit with rapid clearance. Hamsters could be more suitable than mice as a model for SARS-CoV infection (83).

#### Pigs, Chickens, and Ducks

The susceptibility of pigs, chickens, and ducks to supporting SARS-CoV2 replication is very low (72).

The overall assessment of the experimental trials on different animals and birds denotes that animal models that were permissive to SARS-CoV and SARS-CoV2 infections were ferrets, cats, and nonhuman primates. Viral replication in the lower respiratory system of ferrets and cats was very mild (72). However, distinct clinical signs and viral replication in the upper and lower respiratory tract of cynomolgus and rhesus macaques were evident. It was evident that cynomolgus macaques showed more pulmonary signs. Although the severity of SARS-CoV and SARS-CoV2 in nonhuman primates was less than in ferrets and cats, they could be considered as promising animal models to study the pathogenesis of both viruses in the lower and upper respiratory tract.

## The Zoonotic Nature of CoVs

The emergence of new zoonosis is highly influenced by different factors and determinants. The factors and determinants that initiate new zoonotic disease that might end in spillover are of ecological, epidemiological, pathological, and cultural nature (84). The major factors that play a significant role in the emergence of new zoonotic disease will be elaborated on below.

### Factors and Barriers That Interfere With the Emergence of New Zoonotic Diseases

The main way to understand the factors that initiate zoonosis that leads to spillover is to conceptualize the research in quantitative and qualitative relations between these factors (84). For instance, it is clearly understood that anthropogenic factors are one of the major determinants in initiating newly emerging zoonotic diseases; however, behavioral and cultural factors cannot be ruled out in the exacerbation of a newly emerging spillover. The scale of any spillover of newly emerged zoonosis is mainly due to the interaction among the following factors (85):

(1) The dynamic of the disease in reservoir species
(2) The extent of the pathogen exposure
(3) The human susceptibility to infection.

The accumulated evidence emphasizes that the newly emerged zoonosis in recent decades is mainly due to vast anthropogenic changes. The vulnerability of these changes has been exacerbated by behavioral determinants, nutritional and cultural factors related to foodborne zoonotic diseases and pathogen dynamic transmission (84). The exacerbations could be attributed mainly to deforestation, bushmeat hunting, the trade in exotic wild life trade, and demographical changes such as massive urban sprawl (84).

Extensive studies and analysis of the factors that have been referred to as prime inducers of the emergence of new zoonosis are of great importance in developing postulating approaches to predict the possible hotspots for the emergence of new zoonotic diseases (86). The hotspots could be recognized or designated based on the major activities that are prime possible factors in initiating new emerging diseases, such as the considerable increase in bushmeat hunting, the trade in exotic animals, urban sprawl, and/or any significant amendments in wildlife biodiversity. Designing model(s) to predict the factors that foster the emergence of new zoonosis is crucial in considering measures that interfere with pathogen capabilities to breach the barriers standing between the reservoir host and the human host (85, 86). Plowright et al. (85, 86) have anticipated several barriers that pathogens need to breach to be able to get a foothold in humans. The presumed hierarchy of barriers that was built with a cascade effect starts from the distribution of the pathogen from the reservoir host through several barriers such as the intensity of the infection, the extent of the release from the reservoir host, and its prevalence, ending up with adaptation and circulation in humans, which could lead to spillover (85, 86).

### Dynamics and Mechanisms of the Emergence of New Zoonotic CoVs

The diversification of CoVs in regard to their species and intraspecies infection is highly related to the genome structure, mechanisms of replication, and transcription of CoVs (14). The CoVs as RNA viruses exist as quasispecies due to their high rate of mutation, which define their coexistence in wide variants (87). The mutation rates in the RNA of CoVs is estimated at moderate to high. The average substitution rate for CoVs was ~10^−4^ substitution per year per site (13). For instance, the nucleotide mutation rate of the IBV hypervariable region of S gene was estimated at 0.3–0.6 × 10^−2^ per site per year. On the other hand, the substitution rate of the same gene in 229E CoV was estimated at ~3 × 10^−4^ per site per year, whereas the substitution rate in SARS-CoV was estimated at 0.8–2.38 × 10^−3^ nucleotide per site per year (13). Despite the high mutation frequency of the RNA of CoVs, the fidelity mechanism mediated by RNA-dependent RNA polymerase (RdRp) plays a crucial role in maintaining the scale of recombination to preserve the kind of CoVs without jeopardizing their continuous diversity and evolution (87).

The dynamic recombination and mutations of CoVs are mainly at the level of the transcription and replication of RNA synthesis. At the RNA replication, a full-length negative-strand template will be synthesized from the positive RNA. Furthermore, the transcription event is associated by synthesis of several negative subgenomic templates that later generate mRNA. The transcription of the subgenomic RNA templates is carried out by a mechanism termed “the discontinuous RNA transcription model”. This unique and peculiar model is driven by one of the important RdRps, the nonstructural protein 14 exoribonuclease (nsp14 ExoN). The discontinuous RNA transcription mechanism is not fully elaborated. The nsp14 ExoN performs the fidelity by continuous association and dissociation from the negative full-length RNA by recognizing conserved sequences called transcription regulatory sequences (TRS) (87).

Hence, the genotypes expansion and continuous evolution of CoVs are generated through a set of continuous mutations in the viral nonstructural enzymes and recombination with homologous subgenomic RNA templates. Mutation or attenuation in TRS sequences have a major effect on viral transcription and viral replication. However, mutation in nsp14 ExoN, which has a vital role in fostering RNA fidelity during replication and transcription, could drastically cripple the virus (87). The nsp14 ExoN mutants that were generated by site-directed mutations indicated a notable decrease in the fidelity rate during viral RNA replication (87).

In sum, generating new CoV variants is maintained by continuous diversity and evolution through the following mechanisms:

(1) The replication rate is often associated with mutation and recombination due to flexibility in the fidelity mechanism.
(2) Despite the large size of CoV RNA genome in comparison with other single stranded RNA, it is characterized by extra plasticity that enables the virus further modification by mutations and recombination.
(3) The tendency of CoVs to switch templates during RNA replication by a mechanism known as discontinuous RNA transcription with high homologous templates in mixed infections provides an opportunity for possible recombination (14).

## Therapeutical Trials for the Treatment of SARS-CoV2

There is as yet no efficient prophylaxis or treatment for SARS-CoV2 infection. Knowledge of SARS-CoV2 virology is growing rapidly and offers large numbers of potential drug targets. Potential or repurposed medications to treat SARS-CoV2 have previously been used to treat SARS-CoV and MERS-CoV outbreaks with variable efficacy (88, 89). The following are the most common groups of drugs that have been used in the clinical trials in SARS-CoV2 therapy.

### Antimalarials

Chloroquine and hydroxychloroquine have been used since the 1930s to prevent and treat malaria and to treat chronic inflammatory diseases, including rheumatoid arthritis and systemic lupus erythematosus (90). They both have immunomodulatory downregulation for cytokine production and inhibiting the autophagy and lysosomal activity in host cells. They have also been effective at preventing viral entry into cells by inhibiting glycosylation of host receptors, proteolytic processing, and endosomal acidification (91).

### Antivirals

This group of antivirals include the RNA polymerase inhibitors, remdesivir (92), favipiravir (93), and ribavirin (88, 89), which were found to have activity against RNA viruses. Lopinavir/ritonavir oral combination is an HIV drug that appears to have *in vitro* activity against other novel coronaviruses through 3-chymotrypsin-like protease inhibition (94). A neuraminidase inhibitor, oseltamivir, labeled for influenza therapy, has no activity against SARS-CoV2 *in vitro* (95). Arbidol (also known as Umifenovir) has a unique mode of action toward the S protein/ACE2 interaction by inhibiting the viral-membrane fusion (96).

### Adjunctive Agents

Acute lung injury and acute respiratory distress syndrome (ARDS) could be ameliorated by corticosteroids. Tocilizumab, a monoclonal antibodies agent, is also applied to lessen the side effects of ARDS (46). Intravenous immunoglobulin has been also used as a treatment option (97). There is no strong evidence for or against non-steroidal anti-inflammatory drugs (NSAIDs) use in treating COVID-19 patients, and they could be used as a choice for pain management when required. Due to their known risks, NSAIDs should be used cautiously in patients with renal or cardiac diseases or the elderly (98, 99).

In summary, all the therapeutical trials with all of the above drugs were of partial effect against SARS-CoV2 (100). Nevertheless, dexamethasone has been proven to reduce the mortality rate in SARS-CoV2 patients by 29% of those requiring mechanical ventilation and 21% in those requiring oxygen supply (101).

## The Current Situation and Implications for the Future

The scientific community has invested significant effort in the campaign against the SARS-CoV2 pandemic. Despite all the overwhelming knowledge that has been accumulated since the start of the disease, control of this disease or developing an effective cure remains beyond current possible expectations. Lessons should be comprehended from the continuous failure to unravel the wide range of the pathogenicity and epidemiology of animals and bird CoVs.

## Conclusion

CoVs are ubiquitous viruses that circulate in a wide range of mammals, bird species, and human. The diseases inflicted by these viruses range from very mild to severe with high morbidity and/or mortality rates and high losses to the economy. CoVs' most peculiar feature is their mechanism of replication and transcription, which allow generation of variants capable of interspecies and intraspecies infection. TGEV, for instance, which infects dogs, gives rise to a PRCOV variant that circulates in pig herds. The feline CoVs FECV and FCoV are also examples of intraspecies variants.

Despite the elicitation of innate responses that lead to the priming of interferon stimulated genes, the evasion mechanism of CoVs is capable of rendering the innate responses flawed. Cellular immune responses, on the other hand, were shown to be elicited effectively by CoV infection, particularly the CD8^+^ cytotoxic cells. Nevertheless, viral evasion strategies inflict a noticeable setback by initiation of apoptosis. However, ample evidence advocates a long-lasting memory. Animals and bird vaccines for CoVs have been applied extensively in the field, but their efficacy remains questionable, especially FCoVs and IBV vaccines.

The pursuit to designate the susceptible animal models to study the SARS-CoV and SARS-CoV2 pathogenicity continues. Although the infections are restricted to the upper respiratory tract, experimental infection of ferrets and cats indicate advanced clinical signs. Experimental infection of macaques, on the other hand, revealed mild symptoms in the upper and lower respiratory tract. Overall, designating susceptible animal models for SARS-CoV and SARS-CoV2 requires further efforts. Prospects for success could lie in developing genetically modified animal models (induced models). The failure of the therapeutic trials of several drugs to treat SARS-CoV2 in humans emphasizes the need for intensive research in this aspect.

The high recombination and mutation dynamic of CoVs make in-depth research into the factors and determinants that give rise to new emerging zoonotic variants urgent. Extensive investigation into the anthropogenic factors is vital to anticipate the spillover hotspots and to design measures that prevent or encounter the newly emergent zoonosis.

The overwhelming experiences in tackling CoV infection in animals and research in vaccination failure urge close collaboration by interdisciplinary experts by implementation of the One Health concept.

## Author Contributions

All authors listed have made a substantial, and intellectual contribution to the work and approved it for publication.

## Conflict of Interest

The authors declare that the research was conducted in the absence of any commercial or financial relationships that could be construed as a potential conflict of interest.
